# Phase II randomized trial comparing metronomic anthracycline-containing chemotherapy versus standard schedule in untreated HER2 negative advanced breast cancer: activity and quality of life results of the GOIM 21003 trial

**DOI:** 10.1016/j.breast.2024.103725

**Published:** 2024-04-05

**Authors:** Laura Orlando, Evaristo Maiello, Michele Orditura, Anna Diana, Giuliano Antoniol, Maria Grazia Morritti, Michele Aieta, Mariangela Ciccarese, Salvatore Pisconti, Roberto Bordonaro, Antonio Russo, Antonio Febbraro, Paola Schiavone, Annamaria Quaranta, Chiara Caliolo, Dario Loparco, Margherita Cinefra, Giuseppe Colucci, Saverio Cinieri, Gruppo Oncologico dell’Italia Meridionale (GOIM)

**Affiliations:** a Medical Oncology Division, “Antonio Perrino” Hospital, Brindisi, Italy; b Medical Oncology Division, “Casa Sollievo della Sofferenza”, San Giovanni Rotondo, Foggia, Italy; c Medical Oncology Division, AORN Caserta, Italy; d Medical Oncology Division, Ospedale del Mare, Napoli, Italy; e Giulio Antoniol - DGIGL - Polytechnique Montreal, Canada; f Hemato-Oncology Department, CROB-IRCCS, Rionero in Vulture, Potenza, Italy; g Medical Oncology Division, “Vito Fazzi” Hospital, Lecce, Italy; h Medical Oncology Division, “San Giuseppe Moscati" Hospital, Taranto, Italy; i Medical Oncology Division, Garibaldi Hospital, Catania, Italy; j Medical Oncology Division, “Paolo Giaccone” Hospital, Palermo, Italy; k Medical Oncology Division, Ospedale Sacro Cuore di Gesù-Fatebenefratelli, Benevento, Italy; l Medical Oncology Division, Istituto Tumori, Bari, Italy

**Keywords:** Metronomic chemotherapy, Her2 negative advanced breast cancer, Anthracyclines

## Abstract

**Background:**

Optimizing chemotherapy to achieve disease and symptoms control is a noteworthy purpose in advanced breast cancer (ABC). We reported the activity and quality of life of a phase II study, comparing metronomic regimen with standard schedule as first line chemotherapy for ABC.

**Methods:**

Patients with HER2 negative ABC were randomized to non-pegylated liposomal doxorubicin (NPLD, 60 mg/m2 every 3 weeks) and cyclophosphamide (CTX, 600 mg/m2 every 3 weeks) (Arm A) or NPLD (20 mg/m2 day, on day 1, 8 and 15 every 4 weeks) and metronomic daily oral CTX 50 mg (ARM B). Primary end-points were overall response rate (ORR) and quality of life, secondary progression-free survival (PFS), overall survival (OS) and toxicity.

**Results:**

From August 2012 to December 2017, 121 patients were enrolled, 105 evaluable. Median follow-up was 21.3 months. Most patients had hormone receptor positive. ORR was 43 % in arm A and 50 % in arm B. Median PFS was 8.9 months in arm A and 6,4 months in arm B. There was no difference in OS. Total score was not clinically different between the two arms. Grade 4 neutropenia was observed in 12 patients and 16 patients respectively; alopecia G2 in 41 % (77 %) vs 14 (27 %) in arm A and in arm B respectively. One cardiac toxicity was observed (arm A).

**Conclusions:**

First line metronomic chemotherapy for HER2 negative ABC had similar clinical activity and quite better tolerability than standard schedule and could be considered a further treatment option when chemotherapy is indicated.

## Highlights

• Despite the role of biological and targeted drugs, chemotherapy remains a fundamental component of the therapeutic algorithm of ABC.
• The purpose of treatment for ABC is relief of symptoms related tas well the maintenance of a good quality of life.
• Treatment with low doses of anticancer drugs given on a continuous or frequent regular schedule over a long time is called metronomic chemotherapy. Metronomic chemotherapy causes less severe side effects than standard chemotherapy.
• In our study, a metronomic schedule with liposomal anthracyclines is able to achieve similar ORR when compared to standard anthracycline schedule, while maintaining quality of life.
• Toxicity was quite similar in the two arms, however dose delay was less frequent in metronomic arm.

## 1 Introduction

Breast cancer (BC) is the most common cancer and the leading cause of cancer death in women [1]. Systemic chemotherapy with cytotoxic agents had been the mainstay treatment strategy for advanced breast cancer (ABC) for many decades and it is still considered a crucial component of therapies [2]. Currently, there is not generally accepted first-line chemotherapy for HER2 negative ABC and different schedules, combinations and approaches are used in clinical practice.

Anthracyclines and taxanes are the most employed agents for their high activity, leading to an objective response rate of 20–80 % in ABC [3].

The toxicity profile as well as the type of previous (neo)/adjuvant chemotherapy are important factors in determining the optimal choice of a cytotoxic agent or combination after metastases onset [4,5].

Non-pegylated liposomal doxorubicin (NPLD) was developed to overcome the drawbacks associated with non-liposomal formulation. In the Cochrane metanalysis, NPLD was associated with a significantly reduced risk of cardiotoxicity compared with doxorubicin, even in patients previously treated with conventional anthracyclines [6].

NPLD combined to cyclophosphamide has been approved as first line therapy for ABC, based on results of a multicenter trial, in which 291 patients were randomized to receive NPLD plus cyclophosphamide or doxorubicin plus cyclophosphamide. NPLD improved the therapeutic index of doxorubicin by significantly reducing cardiotoxicity and grade 4 neutropenia and provided comparable antitumor efficacy, when used in combination with cyclophosphamide as first-line therapy for ABC [7].

Among strategies to reduce chemotherapy toxicity burden, metronomic chemotherapy (MTC) is one of the most tested and promising. It consists of the frequent, even daily administration of chemotherapeutics at doses significantly below the maximum tolerated dose, with no prolonged drug-free breaks [8]. Preclinical studies have identified the tumor endothelial cell as the main target of MTC, but other mechanisms of action, such as stimulation of immune response, circulating endothelial cells (CECs) inhibition and direct action on tumor cells have been described too [8]. Oral cyclophosphamide was the first and most tested drug in metronomic schedules in breast cancer therapy because of its manageability [9,10].

Patient-reported outcome measures (PROMs) are reports of patient's health condition that comes directly from the patient. The inclusion of PROMS in clinical trials might enhance the understanding of treatment and disease impact on quality of life (HRQoL) [11]*.* One of the frequently used questionnaires for measuring the HRQoL in patients with breast cancer is the Functional Assessment of Cancer Therapy-Breast (FACT-B). FACT-B is a 37-item instrument designed to measure five domains of HRQoL in breast cancer patients: physical, social, emotional, functional well-being as well as a breast-cancer subscale. It consists of the FACT-General (FACT-G) plus the Breast Cancer Subscale (BCS), which complements the general scale with items specific to quality of life in breast cancer [11]. Trial outcome index (TOI) consists of the sum of physical, functional and breast cancer specific subscale [12].

The aim of the GOIM 21003 trial was to compare, in patients with HER2 negative ABC, a metronomic schedule of NPLD and cyclophosphamide to the standard schedule in terms of efficacy, tolerability and quality of life.

## 2 Patients and methods

### 2.1 Study design and statistical considerations

The GOIM 21003 trial is a randomized, two-arms, open-label, multicenter phase II trial, conducted across 9 sites on behalf of Gruppo Oncologico Italia Meridionale (GOIM). Patients were randomly assigned 1:1 to NPLD (60 mg/m2) plus cyclophosphamide (600 mg/m2) both delivered on three weeks schedule (ARM A) or weekly NPLD (20 mg/m2 on day 1, 8, 15 every 28 days) plus oral cyclophosphamide (50 mg daily) (ARM B). Treatment continued until disease progression, unacceptable toxicity, withdrawal of consent, death, or maximum cumulative dose of anthracycline.

Primary end points were overall response rate (ORR, best overall response recorded since the start of treatment until disease progression or recurrence, or death) and quality of life evaluated with FACT-B questionnaires. Secondary endpoints were progression-free survival (PFS, time since randomization until first documented progression of disease or death from any cause, whichever occurs first), overall survival (OS, time since randomization to death from any cause) and toxicity.

The study wanted to verify the null hypothesis that the real effect of the treatment for each schedule was at most 0.15 versus the alternative hypothesis that it is 0.30. Based on Flaming's design, 55 patients per arm had to be enrolled to verify this hypothesis in order to have a power of 90 % with an error α = 0.10. A schedule was considered promising if at least 12 responses were detected in the 55 patients. An interim analysis was scheduled after the first 20 patients enrolled. A schedule with less than 3 responses was considered ineffective. The binomial distribution of the confidence interval (CI) to estimate the real effect of the treatments was constructed according to the Duffy and Santer method. In order to verify the presence of an association between schedule and quality of life we also applied the unpaired two samples Wilcoxon test also known as Wilcoxon rank sum test or Mann-Whitney *U* test. Friedman test was used to detect repeated measurement differences. For PFS, patients without events were censored at the time of the last evaluable tumor assessment, or, if they had no assessment, at the time of randomization assignment +1 day. For OS, patients without follow-up information were censored at the day of last study medication. Patients without postbaseline information were censored at the time of randomization assignment +1 day. Progression free survival (PFS) was measured from the date of assignment to the date of first relapse or the last follow up date without evidence of disease progression.

The distribution of PFS and OS was estimated according to Kaplan Meier's method. The Fisher and χ2 tests were used to compare the baseline characteristics and toxicities between the two arms. A p < 0.05 was considered statistically significant. All patients were included in the analysis except those who had never received treatment. We used Pearson's Chi-square test to determine whether there was a statistically significant interdependence between adverse events and the two schedules.

In all statistical test, we fixed significance level, the probability of rejecting the null hypothesis when it is true, at 0.05. All statistical analyses were performed with the R environment, version 4.1.2, on a 64 bits MacBook Pro.

The toxicity was reported for all patients who received at least one dose of study medication.

The GOIM 21003 trial was conducted in full accordance with the guidelines for Good Clinical Practice and the Declaration of Helsinki. Written informed consent was obtained from each patient. The trial was approved by each Local Ethical Committee (N EUDRACT: 2012-001325-28).

### 2.2 Patients

Patients with histological proven, measurable HER2 negative advanced breast cancer (ABC) untreated with chemotherapy for advanced setting were eligible; previous endocrine therapy were allowed. Measurable lesions were defined by computed tomography (CT), magnetic resonance imaging (MRI) with both diameters ≥1.0 cm; palpation with both diameters ≥2.0 cm; or unidimensional measurable disease ≥1.0 cm.

Prior hormonal therapy in any setting and/or neo/adjuvant chemotherapy were allowed with a cumulative doxorubicin dose ≤300 mg/m^2^ and epirubicin dose ≤500 mg/m2. Normal hematological, hepatic, renal and cardiac [left ventricular ejection fraction (LVEF) within normal limits] function was required. Patients with elevated bilirubin concentration and/or elevated alanine aminotransferase/aspartate aminotransferase were eligible for inclusion if reduced liver function was secondary to liver metastases. Bisphosphonate use at the time of study entry was allowed.

### 2.3 Efficacy and safety assessment

Physical examination, vital signs, hematology, and biochemistry were performed on day 1 of each cycle. For arm B, hematology was repeated on day 8 and 15 of each cycle. Cardiology evaluation with echocardiography or MUGA scan was scheduled every 4 cycles for both arms.

Tumor measurements according to RECIST 1.1 criteria were assessed at baseline and every 12 weeks (±2 weeks) from randomization until first disease progression based on clinical and radiological (by CT scan or MRI) tumor assessments; bone scan was done if clinically indicated.

Adverse events (AE) were recorded and graded using the National Cancer Institute Common Terminology Criteria for Adverse Events (CTCAE) version 4.0, between the first dose of trial medication until 28 days after all treatment discontinuations.

### 2.4 Quality of life evaluation

Quality of life was evaluated through FACT-B, administered on day 1 of each cycle. Patients with at least 2 questionnaires in the first three cycles were considered evaluable for HRQoL. In the final analysis, FACT- B, FACT-G and TOI were evaluated separately.

## 3 Results

**Fig. 1:**
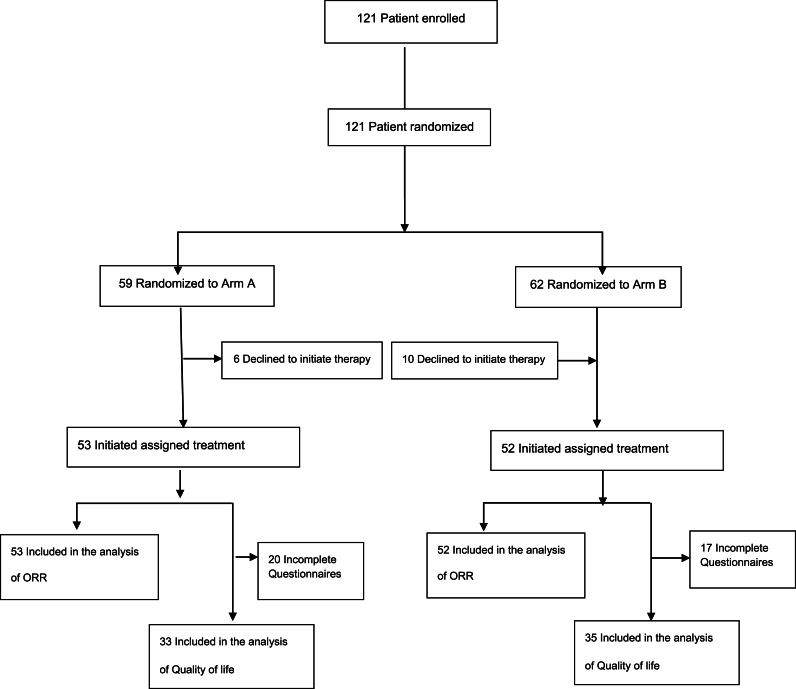
GOIM 21003 trial CONSORT flow diagram.

From August 2012 to December 2017, one hundred twenty-one patients with ABC were enrolled. One hundred five were evaluable for efficacy and safety (Fig. 1). Median follow-up was 21.3 months.

Patients and tumors characteristic are specified in Table 1. Median age was 59 years in both arms. Most patients were postmenopausal. Hormonal receptors were positive in 83 % and 92 % in arm A and arm B respectively. Median number of courses was 6 (range 1–14). Visceral metastases were present in most patients (85 % and 77 % in arm A and Arm B respectively)

**Table 1:** Patients’ characteristics.

Characteristic	GROUP A (n = 53) (%)	GROUP B (n = 52) (%)	p-value
**Median Age(years)**	59 (range 34–75)	59.5 range (35–82)	
**>50**	42 (79)	39 (75)	0.77
≤ 50	11 (21)	13 (25)	
**Met.**			
De novo	24 (45)	19 (36)	0.47
Recurrent	29 (55)	33 (64)	
**DFI recurrent**	Total: 29	Total: 33	
≤24 months	5 (17)	6 (18)	0.999
>24 months	24 (83)	27 (85)	
**Menopausal status**			0.37
Post	39 (74)	43 (83)	
Pre	14 (26)	9 (17)	
**Adjuvant CT**			0.45
Yes	21 (40)	16 (31)	
No	32 (60)	36 (69)	
**Adjuvant ET**			0.28
Yes	25 (47)	31 (60)	
No	28 (53)	21 (40)	
**Adjuvant anthracyclines**	15 (28)	13 (29)	0.67
**Disease location at enrollment**			0.43
No visceral^a^	8 (15)	12 (23)	
- Lymph nodes	6	10	
- Bone	1	9	
- Soft tissue	4	6	
Visceral	45 (85)	40 (77)	
**HR status**			0.23
Positive	44 (83)	48 (92)	
Negative	9 (17)	4 (8)	
**N. of metastatic sites**			0.84
1–3	42 (79)	43 (83)	
≥4	11 (21)	9 (17)	
**Previous ET for MBC**			0.96
Yes	10 (19)	11 (21)	
No	43 (81)	41 (79)	

a Some patients had more than 1 site involved.

**Fig. 2:**
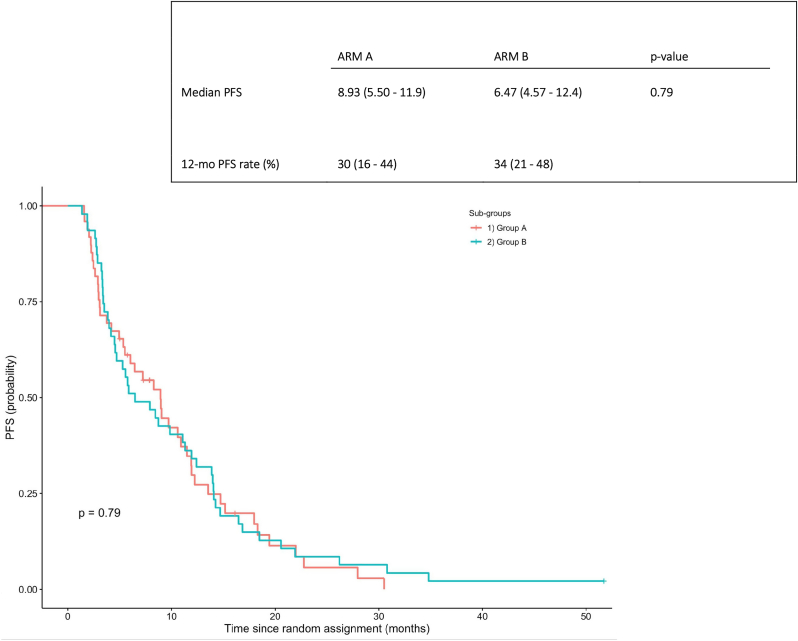
Progression free survival in the GOIM 21003 Trial.

**Fig. 3:**
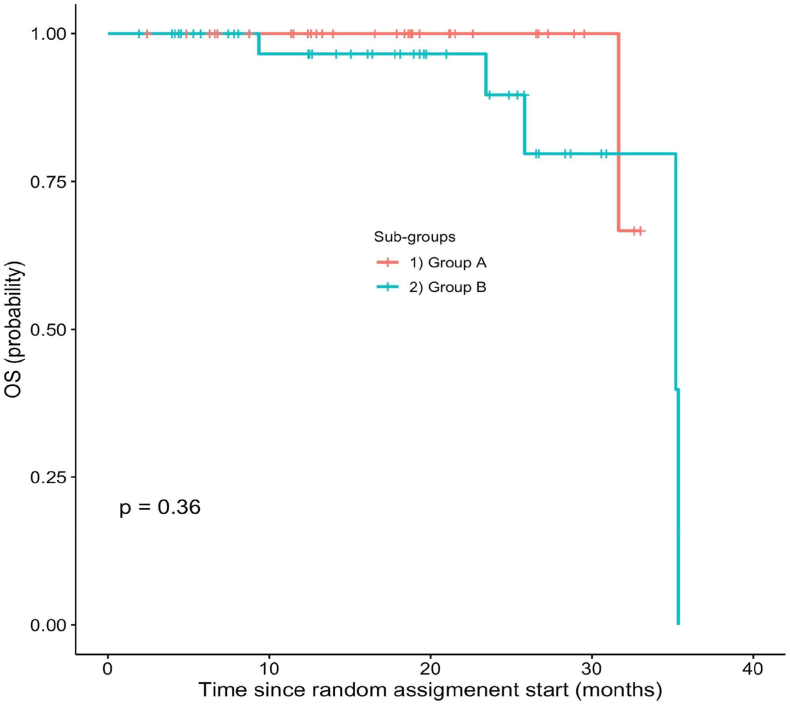
Overall survival in the GOIM 21003 Trial.

ORR was 43 % (95 % CI, 30–58) with 5 CR and 38 PR in Arm A and 50 % (95 % CI, 37–63) with 2 CR and 40 PR in arm B (p-value = 0.6) (Table 2). Median PFS was 8.9 months in arm A and 6,47 months in arm B (Fig. 2). The 12-months PFS was 30 % (range 16–44) in arm A and 34 % (range 21–48) in arm B (Fig. 2). There wasn't difference in median OS (Fig. 3).

**Table 2:** Response according to treatment assignment.

	GROUP A (n = 53) (%)	GROUP B (n = 52) (%)	p-value
CR	3 (5)	2 (4)	
PR	20 (38)	24 (46)	
SD	14 (26)	9 (17)	
PD	16 (30)	17 (33)	
**ORR**	43 (CI 95 %,30–58)	50 (CI 95 %,37–63)	0.6
**CBR**	70 (CI 95 %, 55–81)	67 (CI 95 %,53–79)	0.9

CR = complete response.

PR = partial response.

SD = stable disease.

PD = progressive disease.

ORR = overall response rate.

CBR = clinical benefit rate (ORR + SD ≥ 6 months).

### 3.1 Quality of life

The inclusion criteria for the quality of life analysis was established if at least two questionnaires in the first three cycles were completed. This resulted in a reduction of the population from the initial 121 (total) subjects to 68 (33 in arm A and 35 in arm B, respectively). Non-parametric descriptive analyzes were performed on the populations of the two arms; in particular, the evolution of the medians and the confidence intervals of the medians themselves (95 % interval) were studied; data are reported in Table 3 for the three quality of life indicators.

**Table 3:** Quality of life evaluation according to treatment assignment.

Questionnaire		Cohort		FACT-B		FACT-G		TOI	
		A	B	A	B	A	B	A	B
**I**		32	35	87.5 (71.0–95.0)	89.2 (84.7–99.0)	64.5 (52.1–73.0)	68.0 (63.0–73.0)	55.9 (39.0–64.0)	57.0 (53.1–64.0)
**II**		31	29	89.0 (76.2–94.0)	87.2 (79.3–95.0)	63.0 (54.0–72.5)	64.7 (57.0–76.0)	55.0 (47.0–62.0)	56.0 (50.2–61.0)
**III**		28	29	85.2 (73.0–93.0)	92.8 (81.4–100.0)	62.4 (57.0–67.0)	68.0 (58.0–74.0)	54.5 (41.0–60.0)	57.0 (51.0–65.0)
**IV**		25	21	85.5 (79.0–100.0)	92.9 (82.6–104.0)	65.0 (58.3–77.0)	69.0 (60.0–74.0)	54.0 (51.0–64.5)	61.0 (51.4–67.0)
**V**		22	16	89.9 (78.2–94.0)	94.0 (71.0–106.0)	64.4 (55.2–75.0)	69.9 (56.0–75.0)	55.0 (50.0–60.0)	61.0 (43.0–69.0)
**VI**		21	13	82.5 (67.0–97.5)	79.6 (69.0–98.7)	62.6 (49.0–76.0)	59.0 (47.0–79.0)	55.0 (44.0–63.0)	54.0 (40.0–61.0)
**Overall**		33	35	86.3 (82.5–89.8)	90.0 (86.0–94.5)	74.0 (68.5–77.0)	67.2 (67.2–69.4)	55.0 (53.0–56.7)	57.0 (55.0–60.0)
**Friedman test p -value**				0.39	0.23	0.47	0.16	0.64	0.62
**Wilcoxon test p-value**				0.02		0.0002		0.03	

Quality of life was evaluated through FACT-B, administered on day 1 of each cycle. Patients with at least 2 questionnaires in the first 3 cycles were considered evaluable for HRQoL.

Abbreviations: HRQoL, health-related quality of life FACT-B, The Functional Assessment of Cancer Therapy - Breast, FACT-G, The Functional Assessment of Cancer Therapy - General, TOI, Trial Outcome Index.

As shown in Table 3, baseline mean scores (95 % CI) for all three questionnaires were slightly higher for cohort B rather than cohort A. During treatment we observed a reduction of the values for each arm with a statistically difference in FACT-G between arms in favor of cohort A. However, Friedman tests for FACT-B, FACT-G and TOI were not statistically significant: in no point time, for both arms and questionnaire there was a statistically significant difference between repeated measures. Nevertheless, when data distributions were compared, the Wilcoxon test revealed a statistically significant difference between the two arms. Indeed, median values for FACT-B, FACT-G and TOI tended to be slightly higher in arm A.

### 3.2 Toxicity

Overall, we observed 203 and 188 AE in arm A and B respectively with few grade 3 or 4 toxicities. The most frequent type of AE was myelotoxicity, with neutropenia observed in 64 % in arm A patients and 73 % in arm B patients (G3-4 in 23 % and 31 % respectively) (Table 4). Alopecia, emesis and constipation were the most frequent non-hematologic AE seen in arm A, while asthenia and stomatitis were more frequently observed in arm B. Few grade 3–4 toxicities were reported. In arm A, three patients (6%) experienced a thromboembolic events (one grade 4) and six patients (11%) had dyspnea (5 for treatment-related allergic reactions and 1 for embolic events). Only one cardiac event was observed (grade 4, arm A). The Pearson's Chi-squared test with Yates' continuity correction test comparing grade 3/4 AE frequency between the two treatments has a p-value of 0.92. Frequency of dose reduction was quite similar between the two arms (19 pts in arm A and 17 pts in arm B); however dose delay was more frequent in arm A (49 % vs 36 %).

**Table 4:** Adverse events reported according to treatment assignment.

	Arm A		Arm B	
	AE all grade	3/4	AE all grade	3/4
**LEUCOPENIA**	34 (64)	5 (9)	41 (79)	6 (12)
**NEUTROPENIA**	34 (64)	12 (23)	38 (73)	16 (31)
**Liver toxicity**	4 (8)	1 (2)	6 (12)	1 (2)
**Pyrexia**	4 (8)	NR	6 (12)	NR
**HFS**	1 (2)	NR	1 (2)	NR
**Asthenia**	21 (40)	4 (4)	31 (60)	1 (2)
**Abdominal pain**	5 (9)	NR	6 (12)	NR
**Anaphylaxis**	1 (2)	NR	1 (2)	NR
**Vomiting**	27 (51)	NR	21 (40)	NR
**Constipation**	12 (23)	NR	8 (15)	NR
**Diarrhea**	3 (6)	NR	3 (6)	NR
**Alopecia**	41 (77)	NR	14 (27)	NR
**Stomatitis**	3 (6)	NR	8 (15)	NR
**Dyspnea**	6 (11)	NR	1(2)	NR
**Decreased appetite**	4 (8)	1 (2)	3 (6)	NR
**DVT**	3 (6)	1 (2)	0	NR
**Cardiac toxicity**	1 (2)	1 (2)	0	NR

Abbreviations: AE, adverse event, NR, none reported, DVT, deep vein thrombosis.

## 4 Discussion

Systemic therapy is the mainstay of treatment of ABC. Treatment choice depends on biological features, mainly estrogen receptor (ER), HER2 status and, more recently, PD-L1 expression, germline BRCA1/BRCA2 mutations and PI3KCA mutation [13]. New therapeutic advances opened up treatment options and improved patient outcomes.

In our study, metronomic therapy seemed to have similar efficacy in terms of ORR, PFS and OS compared to standard chemotherapy. Even though the concept of metronomic chemotherapy is relatively old, it has not been robustly evaluated in randomized clinical trials. A great number of studies with MTC are single arm phase II trials and very few trials have randomly compared drugs delivered metronomically with the same drugs delivered at MTD. The majority used all oral metronomic therapy and different intravenous schedule as comparator. The results of a phase III study (METEORA) were presented recently, demonstrating the superiority of metronomic chemotherapy VEC (vinorelbine, cyclophosphamide and capecitabine) over weekly paclitaxel in terms of PFS and time-to-treatment failure (TTF) endpoints [14]. The METEORA study has thus established the VEC regimen as a safe and active option of MCT for the first-line treatment of advanced BC.

Some questions arise from the data of our study. It started when multiple therapeutic options now available to patients had not yet been discovered. Moreover, inhibitors of cyclin-dependent kinases 4 and 6 (CDK4/6) are now an established standard of care for patients with advanced hormone receptor-positive breast cancer and other options for HR positive disease are in process of entering clinical practice [15]. For these reasons, the introduction of chemotherapy in HR positive disease is increasingly delayed. Moreover, in our study very few patients received endocrine-therapy as first-line treatment for their ABC, in contrast with all the International guidelines available [16].

Despite the role of endocrine, biological and targeted drugs, chemotherapy remains a fundamental component of the therapeutic algorithm of MBC. The introduction of new formulation as well as different methods of administration are modalities to improve the risk-benefit ratio for ABC patients. The ORR of 50 % for metronomic arm B documented in this study was higher than the response seen in arm A, despite the absence of statistically significance, and confirmed the clinical activity of metronomic schedules.

In our study, patients with triple negative disease were underrepresented in both arms so it was not possible to evaluate the impact of the two different schedules in that population. The ORR in the HR positive population was 41 % and 52 % in arm A and arm B respectively, confirming the data in the overall population (data not shown).

Data from Victor-6, a retrospective multicenter cohort study, confirmed the role of MTC also in the triple negative breast cancer, reporting a disease control rate in 64.9 % of patients [17]. In our opinion, while taking into account the undeniable role of new therapeutic options (immunotherapy, PARP inhibitors, antibody-drug conjugates), metronomic schedules could be considered in order to avoid some toxicities and to monitor the patient more closely.

Regarding hematologic toxicity, in metronomic arm we unexpectedly observed a greater number of G4 neutropenia; this report might be explained by the weekly monitoring of hematology in arm B (performed on day 1, 8 and 15 of each cycle). In contrast, dose delay were less frequent in arm B, thus allowing the maintenance of dose intensity.

Anthracyclines are among the most widely used agents in early and advanced breast cancer and liposomal formulations allow the rechallenge of anthracyclines used in neo/adjuvant setting. In our study, almost a third of patients have been pre-treated with anthracyclines in early setting, however, only one cardiac event was observed (arm A).

Relief of symptoms related to metastatic lesions as well the maintenance of a good quality of life remain the primary focus of treatment for ABC. HRQoL was the additional primary end-point of our trial. The evaluation of treatment burden using patient-reported outcomes (PROs) supports the overall risk-benefit assessment of a therapeutic regimen and provides information to physician and patient [18]. According to questionnaires reports, patients in arm A seemed to have more modest decline of FACT-B and FACT-G items compared to arm B patients. However, baseline mean scores for all three evaluations were slightly higher for cohort B rather than cohort A, accentuating the differences of the subsequent evaluations. When TOI results were analyzed, no differences were observed between the two arms.

It should be noted that although differences in means and medians are observed, and although these differences are statistically significant, the real clinical value of these differences may be limited. There are two reasons of such a conclusion. The intervals of the means and medians between the two groups substantially overlap. Furthermore, at a percentage level, these differences remain below 9 %. This maximum is reached for the FACT-B in the third cycle.

Some limitations of the quality of life analysis should be stressed. Less than 80 % of patients filled out the questionnaires at all timelines. Moreover, the differential impact of standard three weekly schedule compared to metronomic weekly schedule cannot be highlighted by this analysis, also taking into account the possible impact of more visits to hospital performed by patients in arm B.

In conclusion, the GOIM 21003 demonstrated clinical activity of the metronomic combination of NPLD and oral cyclophosphamide when compared to standard iv schedule in patients with HER2 negative ABC, with mild toxicity, no hair loss and good quality of life.

The future role of metronomic schedules in a continuously evolving scenario of targeted and biological therapies should be more deeply defined. Our study could be hypothesis generating, for further trials designed in different settings such as the endocrine resistant or refractory population.

## CRediT authorship contribution statement

**Laura Orlando:** Writing – original draft, Data curation, Conceptualization. **Evaristo Maiello:** Data curation. **Michele Orditura:** Methodology, Data curation. **Anna Diana:** Data curation. **Giuliano Antoniol:** Methodology, Formal analysis. **Maria Grazia Morritti:** Data curation. **Michele Aieta:** Data curation. **Mariangela Ciccarese:** Data curation. **Salvatore Pisconti:** Data curation. **Roberto Bordonaro:** Data curation. **Antonio Russo:** Data curation. **Antonio Febbraro:** Data curation. **Paola Schiavone:** Data curation. **Annamaria Quaranta:** Data curation. **Chiara Caliolo:** Data curation. **Dario Loparco:** Data curation, Formal analysis. **Margherita Cinefra:** Formal analysis, Data curation. **Giuseppe Colucci:** Conceptualization. **Saverio Cinieri:** Supervision, Conceptualization.
